# A Comparison of Whole Genome Sequencing of SARS-CoV-2 Using Amplicon-Based Sequencing, Random Hexamers, and Bait Capture

**DOI:** 10.3390/v12080895

**Published:** 2020-08-15

**Authors:** Jalees A. Nasir, Robert A. Kozak, Patryk Aftanas, Amogelang R. Raphenya, Kendrick M. Smith, Finlay Maguire, Hassaan Maan, Muhannad Alruwaili, Arinjay Banerjee, Hamza Mbareche, Brian P. Alcock, Natalie C. Knox, Karen Mossman, Bo Wang, Julian A. Hiscox, Andrew G. McArthur, Samira Mubareka

**Affiliations:** 1 Michael G. DeGroote Institute for Infectious Disease Research, McMaster University, Hamilton, ON L8S 4K1, Canada; nasirja@mcmaster.ca (J.A.N.); raphenar@mcmaster.ca (A.R.R.); banera9@mcmaster.ca (A.B.); alcockbp@mcmaster.ca (B.P.A.); mossk@mcmaster.ca (K.M.); 2 Department of Biochemistry and Biomedical Sciences, McMaster University, Hamilton, ON L8S 4K1, Canada; 3 Division of Microbiology, Department of Laboratory Medicine and Molecular Diagnostics, Sunnybrook Health Sciences Centre, Toronto, ON M4N 3M5, Canada; rob.kozak@sunnybrook.ca (R.A.K.); patryk.aftanas@sri.utoronto.ca (P.A.); hamza.mbareche@sri.utoronto.ca (H.M.); Samira.Mubareka@sunnybrook.ca (S.M.); 4 Perimeter Institute for Theoretical Physics, Waterloo, ON N2L 2Y5, Canada; kmsmith@perimeterinstitute.ca; 5 Faculty of Computer Science, Dalhousie University, Halifax, NS B3H 4R2, Canada; finlaymaguire@gmail.com; 6 Peter Munk Cardiac Centre, University Health Network, Toronto, ON M5G 2N2, Canada; hmaan@uoguelph.ca (H.M.); bowang@vectorinstitute.ai (B.W.); 7 Institute of Infection, Veterinary and Ecological Sciences, University of Liverpool, Liverpool L69 3BX, UK; Muhannad.Alruwaili@liverpool.ac.uk (M.A.); Julian.Hiscox@liverpool.ac.uk (J.A.H.); 8 Department of Pathology and Molecular Medicine, McMaster University, Hamilton, ON L8S 4K1, Canada; 9 McMaster Immunology Research Centre, McMaster University, Hamilton, ON L8S 4K1, Canada; 10 Department of Laboratory Medicine and Pathobiology, University of Toronto, Toronto, ON M5S 1A1, Canada; 11 National Microbiology Laboratory, Public Health Agency of Canada, Winnipeg, MB R3E 3M4, Canada; natalie.knox@canada.ca; 12 Department of Medical Microbiology and Infectious Diseases, University of Manitoba, Winnipeg, MB R3T 2N2, Canada; 13 Department of Medical Biophysics, University of Toronto, Toronto, ON M5S 1A1, Canada; 14 Vector Institute for Artificial Intelligence, Toronto, ON M5G 1M1, Canada

**Keywords:** SARS-CoV-2, genome sequencing, bait capture, amplicon sequencing

## Abstract

Genome sequencing of severe acute respiratory syndrome coronavirus 2 (SARS-CoV-2) is increasingly important to monitor the transmission and adaptive evolution of the virus. The accessibility of high-throughput methods and polymerase chain reaction (PCR) has facilitated a growing ecosystem of protocols. Two differing protocols are tiling multiplex PCR and bait capture enrichment. Each method has advantages and disadvantages but a direct comparison with different viral RNA concentrations has not been performed to assess the performance of these approaches. Here we compare Liverpool amplification, ARTIC amplification, and bait capture using clinical diagnostics samples. All libraries were sequenced using an Illumina MiniSeq with data analyzed using a standardized bioinformatics workflow (SARS-CoV-2 Illumina GeNome Assembly Line; SIGNAL). One sample showed poor SARS-CoV-2 genome coverage and consensus, reflective of low viral RNA concentration. In contrast, the second sample had a higher viral RNA concentration, which yielded good genome coverage and consensus. ARTIC amplification showed the highest depth of coverage results for both samples, suggesting this protocol is effective for low concentrations. Liverpool amplification provided a more even read coverage of the SARS-CoV-2 genome, but at a lower depth of coverage. Bait capture enrichment of SARS-CoV-2 cDNA provided results on par with amplification. While only two clinical samples were examined in this comparative analysis, both the Liverpool and ARTIC amplification methods showed differing efficacy for high and low concentration samples. In addition, amplification-free bait capture enriched sequencing of cDNA is a viable method for generating a SARS-CoV-2 genome sequence and for identification of amplification artifacts.

## 1. Introduction

The ongoing pandemic of COVID-19 has infected over 20 million people globally, of which over 750,000 have died (as of 13 August 2020) [1]. COVID-19 is caused by severe acute respiratory syndrome coronavirus 2 (SARS-CoV-2), a novel coronavirus, which emerged in December 2019 [2]. As with any outbreak of a novel pathogen, diagnostics are critical to assess infection in humans and to monitor the extent of the spread of the pathogen. Critical components of outbreak analysis and pathogen identification are second generation high-throughput short-read sequencing and third generation long-read sequencing [3,4]. For COVID-19, the rapid development of diagnostic polymerase chain reaction (PCR) was facilitated by the availability of genome sequences of SARS-CoV-2 isolates [4,5]. In addition, sequencing enables continuous monitoring of circulating strains of the virus to determine any adaptive changes that the virus may accumulate, which may affect its detection, transmissibility, and pathogenicity [6]. Sequencing will also serve an important function as antiviral and vaccine trials roll out, identifying antiviral resistance determinants and vaccine escape mutants, and is essential for detecting viral recombination. For reliable determination of genomic sequences, it is important to have high quality starting genetic material, such as RNA from cultured SARS-CoV-2. Patient samples, such as mid-turbinate swabs, may contain other viruses including seasonal coronaviruses and are also dominated by host genetic material and resident respiratory flora. It is thus imperative to evaluate the performance of genomic amplification and sequencing protocols needed to enhance the derivation of SARS-CoV-2 specific genomic data. Two methods have been widely adopted to obtain SARS-CoV-2 genome sequences from patient samples: (1) the use of SARS-CoV-2 specific PCR primers (tiling multiplex PCR) [7] and (2) the use of bait capture to enrich the SARS-CoV-2 genomic material [8,9,10]. These processes have their own advantages and disadvantages. Tiling multiplex PCR allows for the amplification of numerous viral amplicons but can introduce synthetic artifacts with subsequent cycles. Moreover, divergence from PCR primer sequences can result in suboptimal binding resulting in lost information on genetic diversity or off-target hybridization. Alternatively, bait capture enriches viral RNA by reducing the quantity of non-viral nucleotides, subsequently shrinking the total sequencing volume of the sample. However, the generation of optimal baits requires prior knowledge of the target virus, which is limited in the response against a novel virus. The primary objective of this analysis is to compare genome sequencing results from direct amplification of the SARS-CoV-2 genome (i.e., the Liverpool or ARTIC PCR protocols) [7] with bait capture enrichment from COVID-19 patient swabs with markedly different viral RNA concentrations. Secondarily, we perform a genomic analysis for a) genetic relatedness and b) diagnostic PCR primer mismatch.

## 2. Methods 

### 2.1. Clinical Isolates

Material from mid-turbinate swabs was collected from patients returning from travel during the last week of January and the last week of February 2020. One patient was hospitalized [11] and the other was managed as an outpatient with a less severe disease; both recovered. Diagnostic testing [12] was performed at Public Health Ontario and the results were confirmed at the National Microbiology Laboratory, Winnipeg, Manitoba. This work was approved by the Sunnybrook Institute Research Ethics Board (amendment to 149–1994, 2 March 2020).

### 2.2. Genome Sequencing

Total nucleic acid was extracted from each mid-turbinate swab using the QIAamp Viral RNA Mini kit (Qiagen, Hilden, Germany) without the addition of the carrier RNA. dsDNA for sequencing the library preparation was synthesized using either the Liverpool SARS-CoV-2 amplification protocol^7^, ARTIC SARS-CoV-2 amplification protocol (as described in https://artic.network/ncov-2019) [7], or random priming using the Maxima H Minus Double Stranded cDNA Synthesis Kit (Thermo Fisher Scientific, Waltham, MA, USA) with 2.5 µM random hexamers following the manufacturer’s protocol. For the latter, in a PCR tube 1 µL of Random Primer Mix (ProtoScript II First Strand cDNA Synthesis Kit, NEB, Ipswich, MA, USA) was added to 7 µL extracted RNA and denatured on a SimpliAmp thermal cycler (Thermo Fisher Scientific, Waltham, MA, USA) at 65 °C for 5 min and then incubated on ice. Ten µL 2X ProtoScript II Reaction Mix and 2 µL 10X ProtoScript II Enzyme Mix were then added to the denatured sample and cDNA synthesis was performed using the following conditions: 25 °C for 5 min, 48 °C for 15 min, and 80 °C for 5 min. 

For the Liverpool protocol, primer sequences designed to overlap and amplify the entire SARS-CoV-2 genome in two 15-plex reactions were generously shared by Public Health England. Two 100 µM primer pools were prepared by combining primer pairs in an alternating fashion to prevent amplification of overlapping regions in a single reaction. After cDNA synthesis, in a new PCR tube 2.5 µL cDNA was combined with 12.5 µL Q5 High-Fidelity 2X Master Mix (NEB, Ipswich, MA, USA), 8.9 µL nuclease free water (Thermo Fisher Scientific, Waltham, MA, USA), and 1.1 µL of 100 µM primer pool #1 or #2. PCR cycling was then performed as follows: 98 °C for 30 sec followed by 40 cycles of 98 °C for 15 sec and 65 °C for 5 min. 

For the ARTIC protocol, 1µL Random Primer Mix (ProtoScript II First Strand cDNA Synthesis Kit, NEB, Ipswich, MA, USA) and 1 µL 10mM dNTP mix (NEB, Ipswich, MA, USA) was added to 8 µL extracted RNA and denatured on SimpliAmp thermal cycler (Thermo Fisher Scientific, Waltham, MA, USA) at 65 °C for 5 min and then incubated on ice. 12.5 µL 2X ProtoScript II Reaction Mix and 2.5 µL 10X ProtoScript II Enzyme Mix were then added to the denatured sample and cDNA synthesis performed using the following conditions: 25 °C for 5 min, 42 °C for 50 min and 80 °C for 5 min. After cDNA synthesis, in a new PCR tube 2.5 µL cDNA was combined with 12.5 µL Q5 High-Fidelity 2X Master Mix (NEB, Ipswich, MA, USA). To pool #1 mix 5.87 µL nuclease free water (Thermo Fisher Scientific, Waltham, MA, USA), and 4.13 µL of 10 µM ARTIC version 3 primer pool #1 was added. To pool #2 mix 5.95 µL nuclease free water (Thermo Fisher Scientific, Waltham, MA, USA), and 4.05 µL of 10 µM ARTIC version 3 primer pool #2 was added. PCR cycling was then performed as follows: 98 °C for 30 sec followed by 35 cycles of 98 °C for 15 sec and 65 °C for 5 min.

cDNA synthesis (hexamers only) and PCR reactions (Liverpool amplicons) were purified using RNAClean XP (Beckman Coulter, Brea, CA, USA) at 1.8x bead to amplicon ratio and eluted in 30 µL. Combined ARTIC amplicons were purified at 1.0x bead to amplicon ratio and eluted in 30 µL. Two µL of amplified material was quantified using a Qubit 1X dsDNA HS (Thermo Fisher Scientific, Waltham, MA, USA) following the manufacturer’s instructions. Illumina sequencing libraries were prepared using Nextera DNA Flex Library Prep Kit and Nextera DNA CD Indexes (Illumina, San Diego, CA, USA) according to manufacturer’s instructions. For both Liverpool and random hexamer cDNA libraries (but not ARTIC libraries), half of the prepared libraries were enriched for SARS-CoV-2 using the myBaits Expert Virus SARS-CoV-2 panel (Arbor Biosciences, Ann Arbor, MI, USA) following the manufacturer’s protocol with a 20 h hybridization time at 65 °C and KAPA HiFi HotStart ReadyMix (Roche, Basel, Switzerland) for post-enrichment library amplification, while the other half of each library was sequenced without enrichment. Paired-end 150 bp sequencing was performed for each library on a MiniSeq with the 300-cycle mid-output reagent kit (Illumina, San Diego, CA, USA), multiplexed with targeted generation of ~40,000 clusters per library. A negative control library with no input SARS-CoV-2 RNA extract was included using ARTIC amplification.

### 2.3. Genome Assembly

We developed a complete standardized workflow for the assembly and subsequent analysis for short-read sequencing, released as the SARS-CoV-2 Illumina GeNome Assembly Line (SIGNAL). For the Liverpool and ARTIC amplification-based libraries, sequencing reads pools were combined (as R1 and R2) where needed (i.e., Liverpool amplicons), Illumina adapter sequences were removed and low quality sequences trimmed or removed using Trimmomatic (version 0.36) [13], and then amplification primer sequences removed where needed (i.e., Liverpool and ARTIC amplicons) using cutadapt (version 1.18) [14]. Final sequence quality and confirmation of adapter/primer trimming were confirmed by FASTQC (version 0.11.5) [15]. The percentage of reads derived from SARS-CoV-2 RNA for each library was determined using Kraken2 (version 2.0.8-beta; using RefSeq complete viral genomes/proteins) [16], all non-SARS-CoV-2 reads removed using parsing of HiSAT2 (version 2.1.0) [17] alignments, coverage normalized (samtools mpileup depth of 100,000), and prediction of genome sequenced performed by iVar variant detection (version 1.2, consensus minimum depth = 10) [18]. From these results, assembly statistics were generated by QUAST (version 5.0.2) [19] and depths of coverage were assessed by HiSAT2 (version 2.1.0) alignment of the sequencing reads against the predicted genome sequence [17]. Lastly, sequence variation or coverage gaps in the reads was assessed by BreSeq (version 0.35.0) analysis relative to GenBank entry MN908947·3 (the first genome sequence reported from the original Wuhan outbreak, China) [20]. Separately, sequencing reads were assessed against GenBank entry MN908947·3 using HiSAT2 (version 2.1.0) and visualized using the Integrative Genomics Viewer [21].

### 2.4. Assessment of Clinical Diagnostic PCR Primers

Clinical diagnostic amplification PCR primer sequences were designed in house using Geneious v9.0 (https://www.geneious.com), collated from literature [22,23,24,25,26] and the World Health Organization website [27], and mapped to the MN908947·3 SARS-CoV-2 genome sequence and added as additional reference for BreSeq analysis of the sequencing reads, highlighting any mismatches at PCR priming sites.

### 2.5. Molecular Epidemiology Analysis

To confirm the epidemiological origin of both isolates, the best genome sequence of each was included in a uniform manifold approximation and projection (UMAP) involving the aligned genomes of 8074 SARS-CoV-2 isolates (obtained from Global Initiative on Sharing All Influenza Data, GISAID, https://www.gisaid.org) labelled by country of origin [28]. For UMAP, the approximate genomic differences were estimated using DNA distance determined by the Kimura-80 model of DNA evolution [29], after the removal of the first 55 and last 260 bp of the alignment. The same alignment was used to generate a phylogenetic tree using a RAxML-HPC BlackBox at the CIPRES Science Gateway with GTRGAMMA + I among site rate variation [30]. Both analyses excluded predicted homoplastic sites within the alignment [31].

### 2.6. Data & Software Availability

The SIGNAL workflow is available at https://github.com/jaleezyy/covid-19-signal. Custom software for uniform manifold approximation and projection (UMAP) is available at https://github.com/hsmaan/CovidGenotyper [32]. FASTQ sequences and assembly FASTA have been deposited in NCBI Bioproject PRJNA636446, with assembly FASTA sequences additionally submitted to GISAID (Wuhan-derived: EPI_ISL_413015 as submitted previously, Iran-derived: EPI_ISL_450747). Only sequencing reads that aligned by HiSAT2 (version 2·1·0) to the SARS-CoV-2 MN908947·3 genome were included in the deposited sequence files to avoid the release of sequences derived from patient DNA.

## 3. Result

### 3.1. Clinical Isolates

Two original clinical diagnostic samples from travelers returning to Canada were used for genome sequencing; one from Wuhan, China (“Wuhan-derived”) and one from Iran (“Iran-derived”). Sample RNA, for the generation of cDNA libraries, was extracted from mid-turbinate swabs that were transported in a universal transport medium. The Wuhan-derived sample had a diagnostic qPCR cycle threshold (Ct) value of 31.05 for the envelope (E) gene targets. The Iran-derived sample had Ct values of 18.8 and 20.9 for the RNA-dependent RNA polymerase (RdRp) and E gene targets, respectively.

### 3.2. Genome Sequencing and Assembly

**Figure 1:**
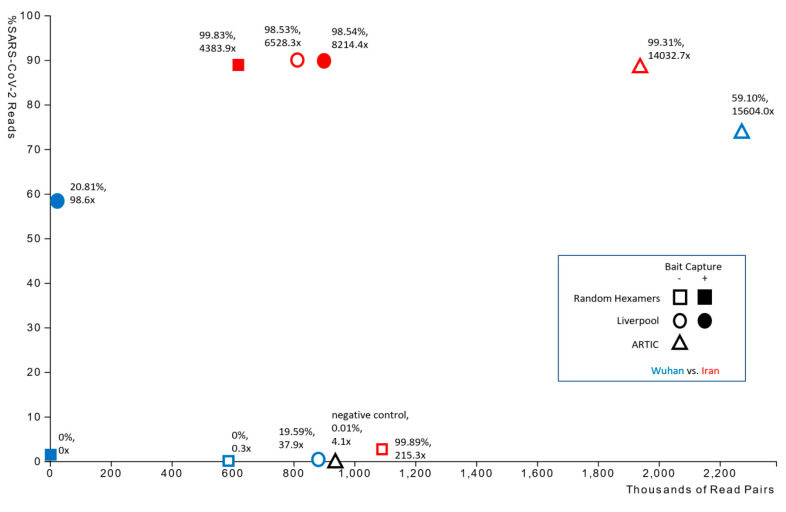
Plot showing the percent of sequencing reads mapping to the SARS-CoV-2 reference genome against the total number of paired reads acquired from each library preparation. Each data point is additionally labelled with a percent fraction and average read coverage of the SARS-CoV-2 genome.

The number of paired reads and percentages of those reads that were derived from SARS-CoV-2 genetic material varied widely between library preparation protocols (Figure 1). In the Wuhan-derived sample, the majority of read data was from the patient genome and therefore resulted in poor SARS-CoV-2 genome coverage and consensus, potentially due to the higher Ct value of the initial sample (i.e., less abundant or fragmented SARS-CoV-2 RNA). By contrast, sequencing data from the Iran-derived isolate consisted predominantly of SARS-CoV-2 molecules and produced a high coverage genome consensus (Table 1). However, ARTIC amplification led to superior results for both the Wuhan- and Iran-derived samples (Table 1), strongly suggesting that the ARTIC protocol would be best for samples with lower viral loads. On examining the sequencing results of the Iran-derived sample more closely, we observed that the Liverpool amplification produced successful results with or without subsequent bait capture enrichment, while cDNA synthesis using random hexamers led to lower relative sampling of SARS-CoV-2 molecules in the sequencing library and poor genome coverage. However, bait capture enriched SARS-CoV-2 cDNA molecules in the sample, producing genome consensus and coverage on par with the Liverpool amplification approaches. None of the sequencing protocols resolved the terminal 5′ and 3′ nucleotide sequences of the genomes, which was consistent with other publicly available sequences (Table 2).

**Figure 2:**
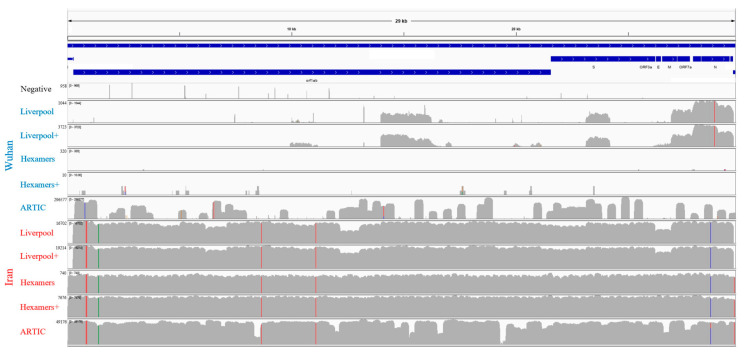
Mapping and semi-log depth of coverage of trimmed sequencing reads for each library preparation against the first Wuhan SARS-CoV-2 genome sequence (NCBI accession: MN908947•3). Y-axis dimensions vary among samples (maximum indicated beside label) and colored positions reflect frequency of SNPs relative to the MN908947•3 genome among the reads (green = A, blue = C, orange = G, red = T). The plus (+) symbol indicates secondary bait capture enrichment. SARS-CoV-2 genome length and organization is highlighted on top.

Examination of read coverage against the first genome sequence reported from the original Wuhan (China) outbreak (GenBank MN908947·3) revealed that despite the better performance of the ARTIC protocol for the Wuhan-derived sample, coverage was highly variable across the genome (Figure 2), with ~10% of locations having less than 100x coverage, ~35% having 100–1000x coverage, and ~53% having >1000x coverage. Amplification of the Wuhan-derived sample using Liverpool primers was limited to a few regions of the genome with 0–100x coverage. This is in contrast to the Iran-derived sample, which had >1000x coverage across >95% of the genome for all methods except the direct sequencing of cDNA (for which ~99% of the genome still had 101–1000x coverage). On average, bait capture enriched the Liverpool amplifications by 1.2 fold and the direct cDNA samples by 19.6 fold, respectively. Although we did not perform secondary enrichment of ARTIC amplification products, these results illustrate that secondary enrichment is not important for PCR amplicons, but valuable for direct sequencing of cDNA. Notably, while ARTIC amplification led to the best overall results for the Iran-derived sample, read alignment revealed several regions with low read coverage (Figure 2), including a 319 bp coverage gap within the *orf1ab* gene (Table 2). This region falls within ARTICv3′s amplicon 64 that has been widely reported to generate little to no sequence coverage [33]. In contrast, the Liverpool amplification produced a more even read coverage across the genome (Figure 2).

Mutation analysis of the well sequenced Iran-derived sample detected one synonymous substitution and four non-synonymous substitutions for the *orf1ab* gene, plus one non-synonymous substitution for the *N* gene (Table 2). While positions 8653 and 28,688 overlap ARTIC PCR primers and could reflect the failed removal of primer sequences by the bioinformatics workflow, both were independently confirmed by the Liverpool amplifications and bait captured cDNA. All five substitutions were consistently supported by 100% of sequencing reads, except for L3606F in the *orf1ab* gene using the Liverpool amplification, the detection of which by BreSeq [20] was obscured by a deletion predicted by a minority of reads; nonetheless iVar [18] consensus generation supported L3606F. This location has been flagged for possible homoplastic sequencing artifacts [31]. Sequencing of cDNA (bait enriched or otherwise) and ARTIC amplification predicted an intergenic nucleotide substitution at position 29,742 in 100% of sequencing reads, yet this was not observed in sequences derived from the Liverpool amplification due to missing read coverage (Figure 2). This position is very close to the polyA tail and while not flagged for exclusion due to poor alignment [31], manual inspection of the read alignments highlighted imperfect mapping of a minority of reads, so this single nucleotide polymorphism (SNP) should be viewed with caution. Finally, both Liverpool and ARTIC amplification methods had minority read support (10.9–22.8% of reads) for a deletion starting at position 11,074 or 11,082, which was not observed for sequencing of unamplified cDNA, but this region has been highlighted for Illumina-specific sequencing artifacts [31].

### 3.3. Assessment of Clinical Diagnostic PCR Primers

SARS-CoV-2 diagnostic PCRs rely on the efficient binding of primers to their designated targets. Mutations in these regions will prevent primer annealing and produce false negative results. Thus, given the critical importance of identifying mutations in diagnostic PCR target sites, our pipeline includes mapping of diagnostic primer sequences [22,23,24,25,26,27] relative to the mutations detected. We identified a number of these have single nucleotide mismatches in the Iran-derived sample, which was supported by 100% of sequencing reads, as well as minority read support for loss of priming sites for the spike protein (Table 2).

### 3.4. Molecular Epidemiology Analysis

**Figure 3:**
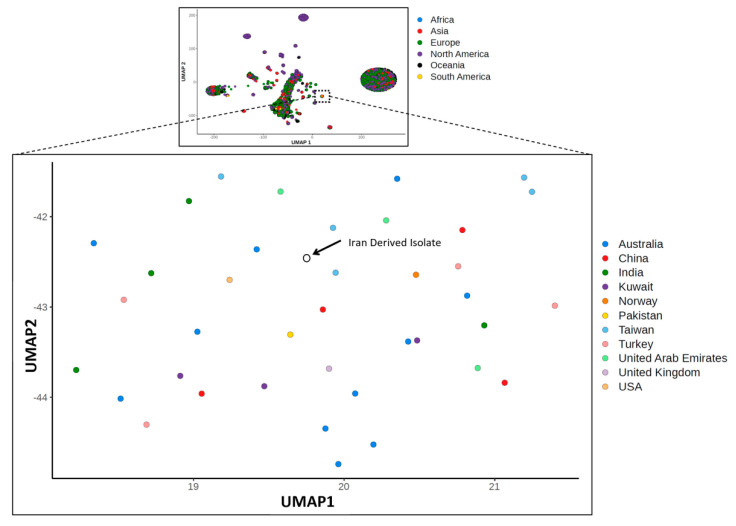
Uniform manifold approximation and projection (UMAP) involving the aligned genomes of 8075 SARS-CoV-2 isolates labelled by country of origin. The Iran-derived sample is indicated by an arrow. The top inset illustrates the analysis of all 8075 isolates, labelled by region, with the zoomed region indicated by the hashed box.

There is very little variation among available SARS-CoV-2 genome sequences, as summarized at GISAID (www.gisaid.org) and exemplified by our own detection of only 6 SNPs between the original Wuhan genome and our Iran-derived sample. By utilizing a uniform manifold approximation and projection (UMAP) [28] of genome sequence similarity, we were able to place this isolate in a small cluster of genomes from Australia (11), China (4), India (4), Kuwait (3), Norway (1), Pakistan (1), Taiwan (5), Turkey (4), USA (1), United Arab Emirates (3), and United Kingdom (1) (Figure 3). Cross-referencing with GISAID metadata revealed that within this small cluster, isolates from Australia (2 isolates), India (4 isolates), and Pakistan (1 isolate) also had travel history associated with the outbreak in Iran. Unfortunately, GISAID did not contain sequences from Iran, but phylogenetic analysis confirmed these UMAP results, placing our Iran-derived sample and the nearby UMAP samples in a well supported clade (Figure S1). The incomplete genome sequence obtained for our Wuhan-derived isolate precluded its inclusion in UMAP and phylogenetic analyses.

## 4. Discussion

Our results underscore the importance of presumptive viral load, based on qPCR cycle threshold, for obtaining a complete SARS-CoV-2 genome sequence, reinforcing the findings of others [34]. While the Liverpool amplification primers provided a more even read coverage of the SARS-CoV-2 genome, amplification using the ARTIC primers was superior for obtaining a complete genome sequence to the point where it was the only successful protocol for one of our samples. Yet ARTIC amplification had regions of low or missing sequence coverage not seen with sequencing of cDNA or the Liverpool amplification (Figure 2). Additionally, low Liverpool and ARTIC coverage at positions ~11,500 to ~13,000 was associated with minority read support for a deletion in the BreSeq analysis, which was not supported by bait enriched cDNA sequencing. This region has been associated with artifacts of Illumina sequencing of amplicons [31]. Yet our standardized iVar-based pipeline (github.com/jaleezyy/covid-19-signal), compatible with and extending the Connor lab ARTIC nextflow pipeline (github.com/connor-lab/ncov2019-artic-nf), was able to overcome these regions of low coverage, favoring the majority reads to generate a final genome sequence. ARTIC amplification and sequencing resulted in a 319 bp gap within the coding region for the *orf1ab* gene (amplicon 64) so this would underpredict any SNPs in this region, while the Liverpool amplification was confirmed to miss a possible intergenic SNP due to missing coverage at the 3′ terminal region of the SARS-CoV-2 genome. Considering the low variation observed to date among SARS-CoV-2 genomes, accurate prediction of every possible SNP using a standardized workflow is of high importance for molecular epidemiological analyses, phylogenetic tree generation, and molecular diagnostic assays. Additionally, it is important for prioritizing virus isolates for subsequent analysis of glycosylation sites and other post-translational modification, as well as cell-culture experiments to investigate in vitro phenotypes. Notably, the prediction of glycosylation sites using NetOGlyc (http://www.cbs.dtu.dk/services/NetOGlyc/) found no differences between the original Wuhan genome (MN908947·3) and our Iran-derived isolate. However, our work did detect mismatches for currently used diagnostics PCR primers, specifically in primers designed by the CDC and the Japanese NID. Clinical laboratories should be aware of this, and we suggest this should be part of ongoing genomic surveillance efforts. We also note that neither amplification method (Liverpool or ARTIC) was perfect, but the results indicated that amplification-free, bait capture enriched sequencing of cDNA is of high utility for the identification of amplification artifacts and may additionally be useful for direct sequencing of SARS-CoV-2 RNA from cell culture. Overall, the availability of alternate protocols permits confirmation of novel mutations by excluding protocol-specific sequencing and analysis artifacts.

Understanding the advantages and limitations of different protocols is essential to population-level whole genome sequencing of SARS-CoV-2 directly from clinical samples. Although the heterogeneity of this source of material may be a limitation, particularly for samples with low quantities and/or quality of RNA, it is the most feasible approach given the constraints of virus isolation. This approach also produces sequences most closely reflecting those within the host. However, we also acknowledge that this work is limited to two clinical samples, which give a preliminary outlook onto the efficacy of each protocol. Additionally, our study only investigated one sample type and evaluation of these protocols with other sample types (e.g., lower-respiratory tract samples) will be informative. Recently, Xiao and colleagues performed comparative studies on sputum, throat swabs, anal swabs, and nasopharyngeal swabs and reported that more viral reads were recovered from nasal swabs than any other sample type [35], although it is not clear if they were using paired samples. This suggests protocol optimization for other sample types is necessary. Overall, standardization and quality controls are necessary for informative broad analyses and to enable DNA sequencing protocol implementation at regional sites of care for enhanced turnaround time to generate actionable data.

## Figures and Tables

**Table 1:** Sequencing read and genome assembly statistics including the total raw read pairs obtained and fraction captured from SARS-CoV-2 RNA, the fraction of 29,903 bp MN908947.3 genome sequence covered, depth of coverage, and number of variants detected relative to MN908947.3.

Sample	Amplification	Enrichment	Number of Paired Reads	Reads from SARS-CoV-2 (%)	SARS-CoV-2 Genome Fraction (%)	Average Depth of Coverage	0–100x Coverage (%)	101–1000x Coverage (%)	>1000x Coverage (%)	# iVar Variants
Negative	ARTIC	No	938,693	0.01	0	4.1x	99.2	0.8	0.1	n/a
Wuhan	Liverpool	No	883,212	0.52	19.587	37.9x	93.88	6.08	0.04	1
Wuhan	Liverpool	Yes	22,119	58.73	20.811	98.6x	89.6	6.8	3.6	1
Wuhan	Hexamers	No	585,396	0.01	0	0.3x	99.9	0.1	0.00	n/a
Wuhan	Hexamers	Yes	1536	1.56	0	n/a	n/a	n/a	n/a	n/a
Wuhan	ARTIC	No	2,271,152	73.86	59.104	15,604.0x	10.6	35.5	53.9	5
Iran	Liverpool	No	813,975	90.13	98.53	6528.3x	1.2	3.1	95.6	6
Iran	Liverpool	Yes	901,124	89.76	98.54	8214.4x	0.7	0.2	99.1	6
Iran	Hexamers	No	1,091,011	2.77	99.89	215.3x	0.43	99.56	0.00	7
Iran	Hexamers	Yes	619,661	89.17	99.83	4383.9x	0.2	0.3	99.6	7
Iran	ARTIC	No	1,935,748	88.25	99.31	14,032.7x	0.2	1.7	98.1	7

**Table 2:** Predicted mutations relative to the MN908947.3 SARS-CoV-2 genome for each library for the high titre Iran-derived sample identified by BreSeq analysis of sequencing reads. Mutations within codons are underlined. All mutations were predicted by 100% of sequencing reads mapping to that position unless otherwise noted. Mutations in bold existed in the final iVar-called genome sequence, while those in italics exist in the final iVar-called genome sequence but were obscured by deletion predictions in the minority reads for BreSeq.

Mutation	Liverpool Alone	Liverpool + Enrichment	Hexamers Alone	Hexamers + Enrichment	ARTIC Amplification	Clinical Diagnostic Primer Mismatch
Unresolved 5′ sequence	259 bp	258 bp	40 bp	0 bp	49 bp	
Unresolved 3′ sequence	200 bp	190 bp	77 bp	139 bp	67 bp	
pos. 835 (orf1ab polyprotein)	F190F (TTC→TTT)	F190F (TTC→TTT)	F190F (TTC→TTT)	F190F (TTC→TTT)	F190F (TTC→TTT)	NIID_WH-1_R854
pos. 884 (orf1ab polyprotein)	R207C (CGT→TGT)	R207C (CGT→TGT)	R207C (CGT→TGT)	R207C (CGT→TGT)	R207C (CGT→TGT)	NIID_WH-1_R913
pos. 1397 (orf1ab polyprotein)	V378I (GTA→ATA)	V378I (GTA→ATA)	V378I (GTA→ATA)	V378I (GTA→ATA)	V378I (GTA→ATA)	
pos. 8653 (orf1ab polyprotein)	M2796I (ATG→ATT)	M2796I (ATG→ATT)	M2796I (ATG→ATT)	M2796I (ATG→ATT)	M2796I (ATG→ATT)	Spike_F1
pos. 9502 (orf1ab polyprotein)			5.0% of reads suggest A3079A (GCC→GCT)			Spike_F1
pos. 11,074 (orf1ab polyprotein)	11.8% of reads suggest a deletion between positions 10,809 and 13,203	11.8% of reads suggest a deletion between positions 10,809 and 13,203			10.9% of reads suggest a deletion between positions 10,809 and 13,203	Spike_F1
pos. 11,082 (orf1ab polyprotein)	18.1% of reads suggest a deletion between positions 10,817 and 10,819	22.8% of reads suggest a deletion between positions 10,817 and 10,819				Spike_F1
pos. 11,083 (orf1ab polyprotein)	*L3606F (TTG* *→* *TTT)*	*L3606F (TTG* *→* *TTT)*	L3606F (TTG→TTT)	L3606F (TTG→TTT)	L3606F (TTG→TTT)	Spike_F1
pos. 19,285–19,603 (orf1ab polyprotein)					319 bp coverage gap (no aligned reads); amplicon 64	
pos. 27,156 (membrane glycoprotein)			5.3% of reads suggest S212C (AGT→TGT)			
pos. 28,688 (nucleocapsid phosphoprotein)	L139L (TTG→CTG)	L139L (TTG→CTG)	L139L (TTG→CTG)	L139L (TTG→CTG)	L139L (TTG→CTG)	2019-nCoV_N3-F
pos. 29,742 (intergenic)	*no coverage*	*no coverage*	G→T	G→T	G→T	

## Supplementary Materials

The following are available online at https://www.mdpi.com/1999-4915/12/8/895/s1, Figure S1: Clade within the larger 8,075 isolate phylogenetic tree containing the Toronto isolate derived from Iran, with isolates associated by UMAP marked by an asterisk (red indicating travel history associated with the Iran outbreak). Branch lengths represent evolutionary distance while node labels represent bootstrap support.
