# Supplementary figures and images for: A Comparison of Whole Genome Sequencing of SARS-CoV-2 Using Amplicon-Based Sequencing, Random Hexamers, and Bait Capture
Source: Viruses. 2020 Aug 15;12(8):895. doi: 10.3390/v12080895 (PMC7472420; doi:10.3390/v12080895)

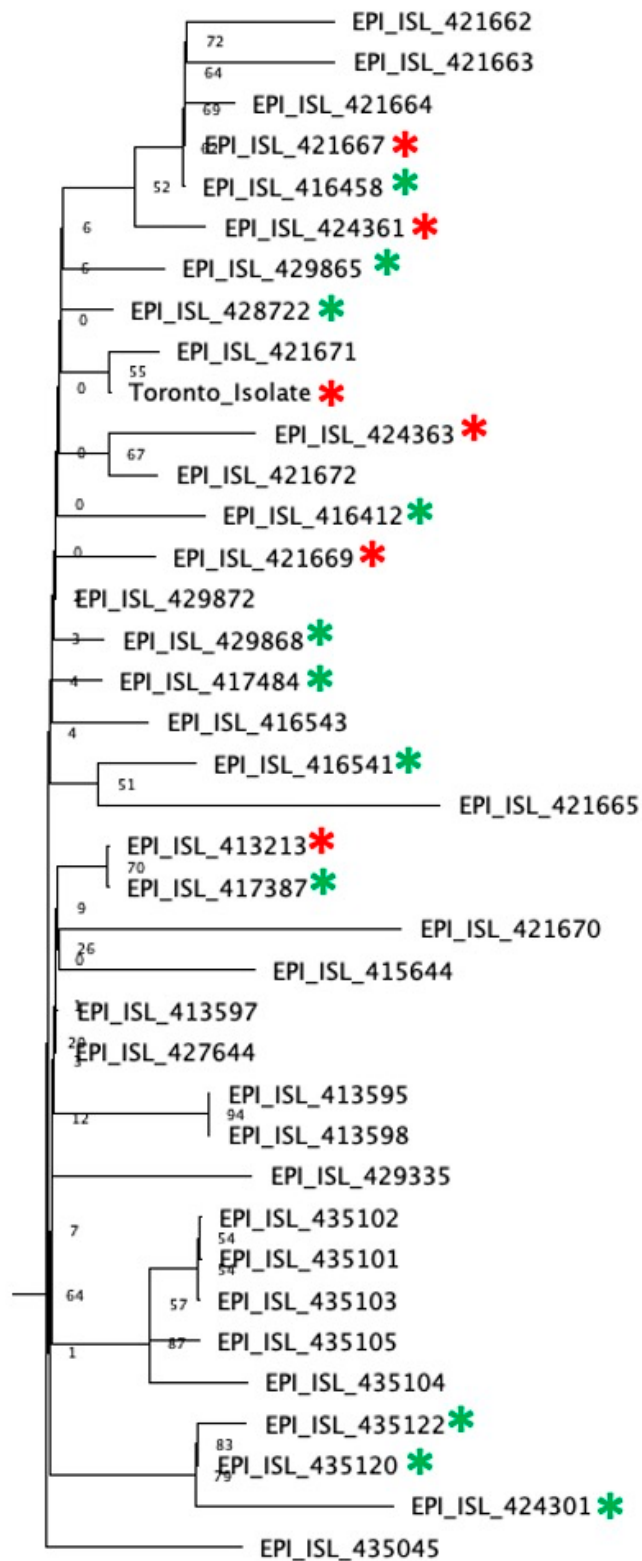

Supplement: Supplementary file 1 [file viruses-12-00895-s001.pdf]
